# Remarks on the Diseases That Appeared in Rutherford County, Tennessee, during the Year 1841, and the Winter of 1841–2; Being a Report Read before the Medical Society of Tennessee, at Its Annual Meeting in May 1842

**Published:** 1842-12

**Authors:** John W. Richardson


ART. II.—*Remarks on the Diseases that appeared in Rutherford County, Tennessee, during the year* 1841, *and the winter of* 1841–2; *being a Report read before the Medical Society of Tennessee*, at its annual meeting in May 1842. By JOHN W. RICHARDSON, M. D.

Amongst the diseases above alluded to, intermitting fever should be first mentioned, because it exists almost continually with us. I have nothing new to communicate on intermittents.

Measles prevailed extensively during the early part of 1841, attended with more arterial excitement than I have usually observed, though nothing of importance transpired in relation to this disease.

Whooping-cough has prevailed for twelve months, attended with its usual characteristics.

Parotitis has also been in my neighborhood for a year, and is still prevailing. It has progressed remarkably slow—remaining in some families for four, and even six months. It is attended with more fever, more cerebral disturbance, and is more often followed by hernia humoralis than I have usually seen it. Some interesting and hitherto unobserved facts were noticed in connexion with this disease. I was requested, last spring, to visit a neighbor, whom I found suffering greatly from pain in the head and swollen testes. No thought was entertained of mumps, as the disease was not in the vicinity at this time. He was treated by venesections, saline purgatives, in which some tartar emetic was dissolved, leeching and warm fomentations to the testes. He soon recovered. Shortly afterwards I saw another case of precisely the same character; and in a few days a third, which was attributed to riding on a wet saddle. The two last cases were treated like the first, and with the same result. In a few days from the attack of the two last patients, parotitis, in its usual form, appeared in each of their families, and two negro men, in one family, were similarly affected with their master. No doubt was then entertained by me but that this inflammation and swelling of the testes was produced by the specific cause of mumps. From that time to the present, parotitis has prevailed in my neighborhood, and many cases have occurred to verify this conclusion. Not one of the individuals who had inflamed testes primarily has had parotitis: but many who had parotitis, had hernia humoralis as a secondary affection. This is common upon exposure. The same treatment succeeded in relieving this secondary affection. Will any of the persons who had testitis primarily, ever have parotitis? I think not. We frequently meet with cases of testitis (I hate the term *hernia humoralis*) in our practice. Scarcely any disease produces so much pain and distress to a man. And here let me remark, that the best anodyne I ever used for it, is just as much "tartar water" as the stomach will retain.

No case of metastasis from the parotids to the mammæ in the female has been observed, though it is frequently spoken of by medical authors; but in several cases under my care the uterus was excited to great action. In pregnant females abortion and miscarriage were produced, and if the translation or sympathetic action took place at the menstrual period, the flow was abundant. There has been no case of death from the disease (parotitis) to my knowledge. In the general it required only rest, light diet, and perhaps one purgative. In many cases nothing was required. The disease has visited almost every family in my region, and four-fifths of the people have had it.

Our summer and autumnal diseases were of the usual character—intermitting and remitting fevers, with cholera infantum. They presented no unusual appearance and yielded to the ordinary treatment. Late in the fall Dr. Murdock and myself attended some rather curious cases of fever, which I will briefly relate. They were all in the same family. One of the cases occurred sooner than the other three, but they were all characterised by the same symptoms, with some slight and unimportant exceptions. The patients were black girls—the oldest nearly grown, and the youngest three years of age. They were attacked with great pain in the forehead, followed by stupor, deficient capillary action, cold extremities, torpid bowels, and a constant disposition to closed eye lids. Reaction was limited and feeble. One of the girls (the oldest) was deaf in one ear, and heard only very loud sounds with the other. She complained of her head, back, and of a choking at the top of the sternum; would frequently rave and talk vociferously when she was aroused. One of the others was entirely deaf for more than a week; pulse quick, frequent and small, occasionally tense; tongue covered with a thick, yellowish-white coat. The duration of their sickness from the time of attack until dismissed from medical attendance, was four weeks. These cases were treated with stimulating diaphoretics, cupping, mercurial purges, blisters and quinine. They all recovered, though convalescence was remarkably slow.

In the month of November an epidemic influenza commenced. Many cases were alarming, so extensive and severe were the bronchial inflammation and pain in the head. These cases were treated with a general bleeding, cupping over the chest, mild purgatives, tartar water, mucilaginous drinks, warm pediluvia, with Dover's powder at night. This disease prevailed through the months of November, December, January, and to some extent in February. The cough was generally very distressing, and frequently remained for a long time after all the other symptoms had subsided. The syrup of squills, blisters and tartar ointment, always avoiding exposure, relieved it.

During the winter we also had some cases of pleurisy, a good many cases of pneumonia, and perhaps a combination of the two, attended with considerable cerebral disturbance. They were treated pretty much in the same manner as influenza, only requiring the lancet more freely, a large dose of calomel and ipecacuanha immediately afterwards, and the constant use of antimonials. Blisters were generally used after active depletion, and with the happiest result.

Cynanche tonsillaris also visited us last winter, attended with high fever and great head-ache. There were not many cases. A free bleeding, followed by the usual antiphlogistic course, and scarification of the tonsils relieved it. If these means were neglected, the tonsils were apt to ulcerate, which required the use of stimulating astringent gargles. Thus in the brief space of twelve months, we have had, in addition to our usual summer and autumnal diseases, measles, mumps, whooping-cough, epidemic influenza, pneumonia, cynanche tonsillaris, and a good many cases of pleurisy. All these diseases, with the exception of measles, prevailed to a greater or less extent through the months of December, January and February; and the mumps and whooping-cough are still with us. We succeeded pretty well in the treatment of these diseases; some cases were violent and required active treatment and good nursing; but to these they generally yielded, and nothing very unusual was discovered in them.

The month of January was a warm and wet month, and towards its close we were visited by an epidemic more unmanageable than any of the former, and more *fatal* than the whole of them combined. The first case that I saw or heard of, occurred on the 24th of January. The disease was characterized by the following symptoms: a chill, or chilliness ushered in the attack; great pain in the head; nausea or vomiting in some cases; more frequently there was neither. In a short time reaction came on (when it came at all), and those who lived to experience it, became either stupid, comatose, or quite vociferous. The great majority became restless, talkative, boisterous, calling those to whom they were most attached in health, pulling and scratching the bed clothes, sometimes biting their finger nails, and occasionally screaming, as though they were frightened. Others lay perfectly stupid; some muttering incoherently, and when aroused, would answer a question rationally, if addressed in a sharp, quick, tone, and would immediately commence writhing and twisting and talking incoherently again. Those who died before any reaction was established, would roll from one side to the other, and toss about in every possible manner, apparently insensible to every thing around them; and even they, when aroused, would sometimes answer rationally.

There were some appearances on the skin of white children that could not be observed in the negro. When the excitement came on, after the chill, a few very red specks were frequently noticed, scattered promiscuously over the forehead, breast, and arms. This eruption was very distinct, and more general in a mulatto girl, than any case I saw. Another eruption of a different character was noted in three patients. Clusters of vesicles (resembling those in shingles) appeared on the lower lip of one patient, extending from the epithelium over the chin. A group of some fifteen or twenty were observed on the cheek of one patient, and as many on the trachea of another.

The eruption first spoken of generally disappeared in a few days without any desquamation, except in one case, where the pimples became larger, more prominent, and filled with a sebaceous looking matter.

Partial deafness in almost every case—blindness, total in one case, almost entire in the second, and partial in a third. In a fourth case there was blindness, I am quite confident, but I could discover no cause for it, as there were no unnatural appearances about the eyes, except a slight contortion. In one of these cases (the first) several whitish flocculi were observed floating, apparently, in the aqueous humor, which continued to increase in size until vision was entirely obstructed. This patient died on the fourth day. In the second case there was the appearance of a beautiful cataract in each eye. The pupils were smartly dilated, and the opacity was not quite large enough to fill the pupil, consequently vision was not entirely obstructed, for I discovered the little fellow could see and distinguish bright objects in an oblique direction. This patient recovered. His eye-sight gradually improved, all morbid appearances were slowly removed, and he sees now as well as he ever did, although he *squints* a little.

In another case, the conjunctiva of the right eye became very red and highly inflamed some two or three days after the patient was seized with the epidemic. It caused him much pain, and vision was entirely destroyed. After the inflammation subsided his eye was minutely examined, and no unnatural appearance was discovered, except a shining yellowness of the crystalline lens or its capsule.

The bowels of all were generally torpid—all the secretions seemed to be arrested, or retained. The urine was frequently not discharged for twenty-four or thirty-six hours after the attack. In one case, the arms of a little girl were paralyzed for several days, and she screamed violently whenever they were moved, or even touched. She recovered. Except the paralysis, this was not a bad case.

In nearly all the bad cases, the head was drawn back on the shoulders, and the whole spine, from the head to the sacrum, was bent like a well strung bow. So great was the extension of the body that many patients could not lie on the back at all. I never saw such a contraction of the spinal muscles, except in the species of tetanus called opisthotonos. The tongue was generally clean at the attack, but after the first excitement it became covered with a short, thick, white coat.

I will relate, briefly, the history of three cases, which will serve as indices to the rest.

Thomas Hardeman, a little boy, aged 12 years, complained of headache on Monday evening. When supper was announced, he was found lying on the bed, and asleep. He was taken up and carried in a gentleman's arms to another room, when he complained of sickness, and vomited freely. Reaction came on directly, and in a short time he perspired profusely.

At sunrise, I was requested to visit him, but did not, on account of the condition of my little daughter, who also had a similar attack, and who died on the 16th day, as a consequence, I believe, of the treatment.

Dr. Starnes, one of my late pupils, who happened to be at my house at this time, visited this patient for me. The Dr. says he found him, immediately after sunrise, cold, almost pulseless, surface of the body covered with large purple spots, rolling on the bed, comatose, and rejecting every thing he swallowed. When aroused, he answered rationally. The most powerful stimulants, hot applications and frictions, sinapisms, a blistered stomach, camphorated ether, an attempt to bleed, &c., all failed to produce reaction, and the little fellow soon expired.

Harriet, a servant girl of my neighbor, J. Sykes, Esq., about the same age, slept quietly through the night, and arose well, to all appearance, in the morning. She took the infant from her mistress, and while opening the door to leave the room, fell to the floor with a groan. Upon examination by her master, he says he found her cramped all over, and speechless Her extremities became cold directly, when she was placed in a hot bath, and an emetic given, according to previous advice. Just after this, and while the emetic was still operating, I saw her. Reaction was being slowly established; it was assisted to a point deemed sufficient, when she was cupped freely between the shoulders, and afterwards bled from the temples. A large dose of calomel was given, and another left for her at night with Dover's powder; spts. mindereri to be given as a febrifuge. The medicine failed to operate on her bowels, and enemata were employed. After a while they succeeded.

She spent a restless night, and I found her next morning in pretty nearly the same condition as when I left her, twisting and turning on her bed, calling her master, and talking incoherently. A blister was applied to the nape of the neck, and very nearly the same course of treatment pursued as will be related in the next case. She died on the fourth day.

Mary, aged 13 years, servant girl of Capt. J. Smith, had a chill, and severe headache. An emetic was given. Reaction came on, attended with great stupor. She was bled to a pint and immediately afterwards I saw her. She had also taken some purgative pills, but they had not operated. She was resting quietly, very stupid, though answering rationally when aroused. Cupped her freely on the back of the neck, applied a blister, and gave 20 grs. calomel. By the next day the medicine had operated several times, and the last discharge was very thin and yellow. Pulse small and quick. Gave a dose of laudanum (20 drops). She complained greatly of her head and back; placed her in a barrel of hot water, and her pulse improved. Gave her 10 grains of blue mass, and 8 grains of Dover's powders, to be taken at night.

The next day her head was drawn back, and the spine curved like an arch.* Pulse as yesterday. Placed her in the bath again—gave a cordial. Discharges from her bowels frequent and small, though of a tolerably good character. Skin dry; tongue covered with a thick, short, white coat. Gave

* The girl Harriet was in the same condition.

her spts. mindereri every hour, and blue mass and Dover's powder at night.

The next day (20th February) found her condition about the same as previously. Exchanged the spts. mindereri for camphorated ammonia, as the patient seemed more feeble. Applied half a dozen dry cups on the spine. Continued blue mass and Dover's powder. Directed the spine to be well rubbed with brandy, cayenne pepper and camphor, and the dry cupping to be repeated at night—to take oil in the morning if the bowels are not moved.

21. Visited patient at 1 o'clock, P. M. Oil had been taken and had operated. Same treatment continued and the bath repeated.

22. Condition, perhaps, a little improved. Same treatment continued.

23. Patient seemed evidently better, and from this time she slowly recovered.

The respiration, in nearly all the cases which I saw, was free; frequently, remarkably soft and easy. In a few cases there was sighing occasionally. There was no cough—no signs of pulmonary engorgement, nor was there any evidence of primary disease any where save in the brain, spinal cord, or ganglionic system. And in proportion as one or the other of these systems was affected, so was the predominance of the symptoms peculiar to it. Was the primary lesion in the brain? There was coma, at times listlessness, pale and vacant countenance, and if the patient was elevated, nausea and vomiting were induced. Was the spinal cord the first to suffer? A tetanic spasm of the cervical and spinal muscles, great pain in the neck and along the whole course of the spine, were the predominant symptoms. Was the ganglionic system attacked? The bowels were torpid, all the secretions suppressed, and medicines had but little effect. The greater number of my cases was a complication of the spinal and ganglionic forms. Severe pain in the head or back was universally complained of by all those who could give any account of their sufferings. Many cases of influenza, and some of pneumonia and tonsilitis "passed off" for this singular disease. But none of the cases which I saw had a single symptom peculiar to the epidemic except pain in the head. I heard of many cases and saw some of those that were called "this head disease," but they were not. The attack came on with a chill, followed by considerable reaction. The determination was to the mucous surface of the lungs, inducing a bronchitis; or to the parenchymatous portion, when a pneumonia was the consequence, and the cough succeeded the reaction in both instances.

The only description, which I have seen, of any form of disease that assimilates this epidemic, appeared in the Gazette Medicale and subsequently in the Medico-Chirurgical Review (No. 86, for 1841). The disease prevailed at Versailles, Rochfort, Metz, and at Strasbourg. The history of the epidemic, as it appeared at Strasbourg, is given by Dr. Wunschendorff; and as it may not be in the hands of all those who read this article, I have concluded to copy it from the Review.

"The *symptoms* of this epidemic meningitis were usually sudden headache, vertigo and vomiting; then delirium, convulsions, and tetanic spasms of the jaw and trunk; and lastly coma, paralysis of the sphincter and other parts of the body, and the usual phenomena of malignant typhus. The bowels were in most cases constipated at first and afterwards became relaxed. The pulse was often remarkably slow at the commencement and in the early stage of the disease. In the majority of cases an herpetic eruption of the lips made its appearance.

According to the predominance of certain symptoms, the Dr. describes an inflammatory, a neuralgic, a convulsive, a delirious, and a comatose form of disease. But he has not been able to point out a connection between these different forms and any special modification of the necroscopic phenomena: on the contrary, the morbid lesions discovered on dissection were almost always nearly the same. The *course* of the disease was very irregular; in many cases it exploded with symptoms of high excitement, under which the patient sunk, or which were succeeded by torpor and various other phenomena. The duration of the disease also varied much in different cases; in some it proved fatal in a few days, or even in a few hours, while in others it was lengthened, after the first violence passed away, to one or several months; before terminating in death, or in a slow and painful convalescence.

The diagnosis was occasionally difficult, the disease assuming the character either of malignant fever, follicular enteritis, or apoplexy. The prognosis was necessarily unfavorable. One half of the patients died; but this mortality is common to this epidemic with sporadic meningitis, which usually proves fatal in two out of every three cases. In the civil hospital at Strasbourg, 21 patients died out of 40 that were admitted; and in the military hospital the mortality was still greater—104 out of 176.

The *treatment* followed was entirely antiphlogistic. The patients were usually bled several times, and at short intervals, varying according to the severity of the symptoms during the early stage. Leeches were applied in great numbers to the temples and behind the ears, and cupping also along the spine was practised freely. Cold lotions were applied to the head, and in some cases mercurial ointment was smeared on the shaved scalp.

Having lost several patients, to whom calomel in large doses was administered, and as there were usually abdominal symptoms present, Professor Forget subsequently discontinued the use of intestinal derivatives, and trusted altogether to mild aperient enemata.

In the more advanced stage of the disease much reliance was placed on cutaneous irritants. At this period, opium in the form of syrup seemed decidedly useful in mitigating the headache and delirium. All stimulant and tonic medicines were hurtful, except during convalescence."

Such is the history of this epidemic. No *cause* has been assigned for it. What relationship it bears to the epidemic that constitutes the principal part of this essay, is left for the decision of those who may examine both as here related. That there are some striking similarities will be discovered by the superficial observer.

No one, so far as I am informed, has ventured to give "the epidemic" either a name or habitation. Nor do I believe any one has promulged, even what he might *suppose* to be the cause. The cause and pathology of the disease are subjects of interest and importance; and as we have no knowledge of either, I will give you the *best faith* I have upon the question, and await further revelation.

The last winter was remarkable for its mildness. The month of January was wet and very warm. Inflammatory diseases had been, and were still prevailing up to the appearance of the epidemic. Why so great a disposition to cerebral, cerebro-spinal and nervous lesions should exist at this time, we are unable to state. For, even if we suppose that the epidemic was not the effect of a peculiar and specific cause, but was only produced by the general cause or causes then acting in the production of the inflammatory diseases which existed at the time of its appearance, still we must conclude that something more was necessary in order to the developement of such peculiar symptoms. Whether the wet and warm month of January was sufficient to produce these peculiarities, is also a question which deserves attention; or whether a cause independent of those then acting, and one *sui generis*, is another question. I am inclined to believe that the cause was peculiar, and produced in every case, where its effects were cognizable, the same symptoms, either of a milder or severer grade. How the cause was generated, I cannot tell. I *believe* it was atmospheric. Some one may say that this is not sufficiently definite. True, it is not as much so as we all would wish; but still it is as definite as our present limited knowledge of various other causes producing peculiar epidemics will justify. A field for speculation is here opened, but as this is not the place nor the occasion for such an enterprise, we will not enter upon it.

Upon the pathology of the disease we would also speak with much caution. The cerebral symptoms were very striking, peculiar, and uncommon. The spinal were not so much so, having several times seen similar symptoms in spasmodic complaints. In the ganglionic system there was evidence of great derangement; medicines had but little, and frequently no effect at all, so completely was that beautiful and important chain, which connects every organ, and holds them in friendly action, unlinked.

The form of cerebral disease in some patients was phrenitis; and in others (the greater number) it was arachnitis. The symptoms immediately preceding death, were very much like those observed in children who die of acute arachnitis terminating rapidly in effusion. The fever was of a typhus form, and the *eruption,* so far as I have observed, is confined to this form of fever.* Bleeding was generally practiced after the manner given in the cases reported, and I cannot say that it always afforded even partial relief. Yet we could scarcely keep our lancets dry in the treatment of such cases.

Emetics at the onset always afforded relief, I thought, and I have reason to believe frequently arrested the disease at once. They had to be given, though, during the headache which preceded the chill. Purgation was of great advantage during the whole course of the disease.

The warm bath had a good effect in all such cases as the second one reported.

Dry cupping and friction, with stimulating articles along the spine, always afforded relief. Patients would frequently request those remedies to be reapplied, when it appeared to me that a *further* use of them would produce much pain.

Blisters to the nape of the neck afforded also considerable mitigation of the cerebral symptoms.

Of all the remedies, however, which I used in the treatment of the disease, I think I derived more benefit from opium than any other one article. It had a good effect, given in any form and almost at any time.

Note.—I have lately had one of the patients, who had this epidemic, under treatment for *deafness* induced by the disease. She could talk very well before she was attacked—did not have a very severe attack—recovered well, but lost her hearing. I have failed in restoring it. She has, in consequence of deafness, forgotten how to talk. She is four years old.

---

* Since writing the above, I have seen a very interesting case of typhoid fever, in which the same eruption appeared very thick on the breast, back, neck and superior extremities.—*August*, 1842.

